# Quantitative Analysis of Cellular Proteome Alterations in CDV-Infected Mink Lung Epithelial Cells

**DOI:** 10.3389/fmicb.2017.02564

**Published:** 2017-12-22

**Authors:** Mingwei Tong, Li Yi, Na Sun, Yuening Cheng, Zhigang Cao, Jianke Wang, Shuang Li, Peng Lin, Yaru Sun, Shipeng Cheng

**Affiliations:** State Key Laboratory for Molecular Biology of Special Economic Animals, Institute of Special Economic Animal and Plant Science, Chinese Academy of Agricultural Sciences, Changchun, China

**Keywords:** Canine distemper virus (CDV), Mink lung epithelial cells (Mv.1.Lu cells), Isobaric tags for relative and absolute quantitation (iTRAQ), proteomics, NF-κB signaling

## Abstract

Canine distemper virus (CDV), a paramyxovirus, causes a severe highly contagious lethal disease in carnivores, such as mink. Mink lung epithelial cells (Mv.1.Lu cells) are sensitive to CDV infection and are homologous to the natural host system of mink. The current study analyzed the response of Mv.1.Lu cells to CDV infection by iTRAQ combined with LC–MS/MS. In total, 151 and 369 differentially expressed proteins (DEPs) were markedly up-regulated or down-regulated, respectively. Thirteen DEPs were validated via real-time RT-PCR or western blot analysis. Network and KEGG pathway analyses revealed several regulated proteins associated with the NF-κB signaling pathway. Further validation was performed by western blot analysis and immunofluorescence assay, which demonstrated that different CDV strains induced NF-κB P65 phosphorylation and nuclear translocation. Moreover, the results provided interesting information that some identified DEPs possibly associated with the pathogenesis and the immune response upon CDV infection. This study is the first overview of the responses to CDV infection in Mv.1.Lu cells, and the findings will help to analyze further aspects of the molecular mechanisms involved in viral pathogenesis and the immune responses upon CDV infection.

## Introduction

Canine distemper virus (CDV), a negative-sense, single-stranded RNA virus, belonging to the genus *Morbillivirus*, family Paramyxoviridae, causes a severe highly contagious lethal disease in carnivores, such as dogs, lions, ferrets, raccoon dogs, foxes, and minks (Williams et al., 1988; Deem et al., 2000; Martella et al., 2010; Zhao et al., 2010; Viana et al., 2015). The disease is distributed worldwide and is characterized by respiratory and gastrointestinal tract symptoms with generalized immunosuppression (Blancou, 2004; Decaro et al., 2004). The immune system dysfunction of CDV infection favors opportunistic secondary pathogens, resulting in high morbidity and mortality in a wide range of carnivore species (Appel et al., 1982; Kauffman et al., 1982; Blixenkrone-Moller, 1989). Generally, in domestic dogs, CDV establishes a systemic infection, initiating transmission from immune cells, such as alveolar macrophages and/or dendritic cells, of the upper respiratory tract to the local lymphatic tissues by immune-mediated progression, and ultimately propagates to most organs and tissues, including epithelial tissues via cell-associated viremia (Appel et al., 1982). Epithelial cells are susceptible to CDV infection and play a role in transmission during the late stages of CDV pathogenesis (Pratakpiriya et al., 2012; Noyce et al., 2013). The virus is amplified and secreted from the epithelial cells of the respiratory, gastrointestinal, and urinary systems of the infected host (Ludlow et al., 2014). The infection of various viruses has been demonstrated to interact widely with numerous host cell proteins. Some interactions elicit changes in the host proteome, as illustrated by the capacity of the virus to both induce and evade the host immune response (Kash et al., 2006), effecting autophagy and apoptosis (Ludwig et al., 2006; Gunnage and Munz, 2009). For measles virus (MV), another morbillivirus closely similar to CDV, cell cycle arrest in lymphocytes (Naniche et al., 1999) and apoptosis in T lymphocytes (FugierVivier et al., 1997) have also been reported. Many studies have reported the effects of CDV infections on the host cell proteins, such as inhibiting STAT1 and STAT2 nuclear import (Rothlisberger et al., 2010), inducing cytokine responses in PBMCs (Nielsen et al., 2009), and inducing lymphocytes apoptosis (Kumagai et al., 2004). However, most of these reports have primarily investigated a single host cell protein or partially selected proteins and the mechanisms of CDV pathogenesis and immunomodulation have not been fully elucidated. Thus, a new approach for further understanding the pathogenic mechanism and immunomodulation of CDV infection is needed, and the identification of global host cell proteins that interact with CDV infection represents one option. More details associated with host responses to CDV infection should also shed some light on potential targets for antiviral agents.

For decades, proteomic assays have been applied as significant tools to analyze the interaction of host responses to viral infection. Investigation of the changes in the proteome upon virus infection is becoming an effective instrument for providing potential targets for antiviral research. This approach has revealed the specific insights into the cellular mechanisms involved in viral pathogenesis for several viral pathogens, including transmissible gastroenteritis virus (TGEV) (An et al., 2014), human influenza A (Vester et al., 2009), canine parvovirus (CPV) (Zhao et al., 2016), marek's disease virus (MDV) (Chien et al., 2012) and infectious bronchitis virus (IBV) (Emmott et al., 2010). Isobaric tags for relative and absolute quantification (iTRAQ) combined with LC–MS/MS analysis have emerged as a powerful quantitative proteomic technique, which has been used for various virus-host interaction studies (Zhang et al., 2009; Liu et al., 2013; Luo et al., 2014).

The present study is the first global view of the changes in the mink proteome upon CDV infection. Based on iTRAQ combined with LC–MS/MS, a quantitative proteomic analysis was performed to identify differentially expressed proteins (DEPs) in mink lung epithelial cells (Mv.1.Lu cells) infected with CDV at 24 hours post infection (hpi). These findings will help to analyze further aspects of the molecular mechanisms involved in viral pathogenesis and systematically understand the host immune responses challenged by CDV infection.

## Materials and methods

### Cell culture and virus infection

Mink lung epithelial cells (Mv.1.Lu cells) were purchased from the Type Culture Collection of the Chinese Academy of Sciences (Shanghai, China) and grown in Minimum Essential Medium (Gibco ®Invitrogen, U.S.A.), supplemented with 10% fetal bovine serum (Invitrogen) at 37°C and 5% CO_2_. The canine distemper virus strain CDV-PS (GenBank accession no. JN896331), a low passage isolate (<7 passages) from a morbid dog in 2013 (Yi et al., 2013), was preserved in our laboratory. The virus was propagated in Vero cells. In the study, three additional passages of the virus were performed in Mv.1.Lu cells, resulting in the virus suspension with a titer of 10^3.1^ TCID_50_/mL determined by a 50% tissue culture infectious dose (TCID_50_) assay (Yamaguchi et al., 1988). Briefly, monolayers of Mv.1.Lu cells in 96-well plates were infected with a 10-fold serial dilution of the supernatant fluids and further incubated for up to 120 h. The wells were assessed for cytopathic effects (CPE) after 3–5 days, and the TCID_50_ was calculated using the Reed-Muench formula. Because of the low virus titer and the impurity of the virus suspension, virus concentration and purification were performed to improve the virus titer and avoid the effect of non-viral components. The clarified suspension was concentrated by polyethylene glycol 6,000 precipitation and purified by ultracentrifugation in a gradient of sucrose according to standard procedures. Sucrose-purified viruses were then titrated using the TCID_50_ assay as described above, and the titer of the virus stocks increased to 10^6.9^ TCID_50_/mL. The attenuated CDV vaccine CDV_3_ strain was treated the same as PS. The virus stocks were aliquoted and stored at −80°C until further use in the following experiments.

For the establishment of viral kinetics, Mv.1.Lu cells were grown in 6-well plates and subsequently challenged by the virus (PS) at a multiplicity of infection (MOI) of 2, calculated based on the infectious virus particle concentration determined as TCID_50_. At 6, 12, 24, 36, 48, 60, and 72 hpi, viral propagation was confirmed by observation of the CPE and viral replication and production of PS nucleoprotein for the different time points analyzed was tested by anti-CDV NP antibody. The one-step growth curve, indicating the viral load with the time, was generated according to Chuzo ushimi with slight modifications (Ushimi et al., 1972). Briefly, 200 μL of culture medium was collected at indicated time, followed by the extraction of total RNA from all samples. qRT-PCR was then applied to detect the viral RNA at each indicated time. For iTRAQ labeling, Mv.1.Lu cells were grown in T75 flasks to 70–80% confluence and subsequently infected with the virus (PS) at an MOI of 2. As an uninfected control, a mock-infection was performed. The cells were collected at 24 hpi for the protein extraction. Three biological replicates were prepared for all samples. All experiments were performed under Biosafety Level 2 conditions.

### Protein isolation, digestion, and labeling with iTRAQ reagents

The collected cells were lysed in lysis buffer containing a protease inhibitor cocktail. The lysate was sonicated and centrifuged at 14,000 g for 40 min, and the supernatant was quantified with the BCA Protein Assay Kit (Bio-Rad, U.S.A.). Subsequently, 200 μg of protein for each sample was digested with 4 μg of trypsin (Promega, WI) overnight at 37°C. According to the protocol of the iTRAQ reagents (8 plex, Applied Biosystems), 100 μg of peptide mixture from each sample was labeled follows: the three mock-infected samples were each labeled with iTRAQ 113, 114, or 115, and the three PS-infected samples were labeled with iTRAQ 116, 117, or 118. The labeled samples were then mixed and dried with a rotary vacuum concentrator.

### Peptide fractionation and LC-MS/MS analysis

To reduce the complexity of the peptide mixtures, iTRAQ-labeled peptides were fractionated by SCX chromatography using the AKTA Purifier system (GE Healthcare). Briefly, the dried peptide mixture was reconstituted and acidified with buffer A (10 mM KH_2_PO_4_ in 25% of ACN, pH 3.0) and loaded onto a PolySULFOETHYL 4.6 × 100 mm column (5 μm, 200 Å, PolyLC Inc., U.S.A.). The peptides were eluted at a flow rate of 1 mL/min with a gradient of buffer B (500 mM KCl, 10 mM KH_2_PO_4_ in 25% of ACN, pH 3.0). The elution was monitored by absorbance at 214 nm, and fractions were collected every 1 min. A total of 15 fractions were collected with screening, and then desalted on C18 Cartridges (Empore™ SPE Cartridges C18 (standard density), bed I.D. 7 mm, volume 3 mL) and concentrated by vacuum centrifugation.

Each fraction was injected for nanoLC-MS/MS analysis. The peptide mixture was loaded onto a reverse phase trap column (Thermo Scientific Acclaim PepMap100, 100 μm^*^2 cm, nanoViper C18) connected to the C18-reversed phase analytical column (Thermo Scientific Easy Column, 10 cm long, 75 μm inner diameter, 3 μm resin) in buffer A (0.1% Formic acid) and separated with a linear gradient of buffer B (84% acetonitrile and 0.1% Formic acid) at a flow rate of 300 nL/min controlled by IntelliFlow technology. The LC-MS/MS analysis was performed on a Q Exactive mass spectrometer (ThermoFisher, U.S.A.) coupled to the Easy nLC chromatography system (ThermoFisher, U.S.A.). The mass spectrometer was operated in positive ion mode. MS data was acquired using a data-dependent top 10 method, dynamically selecting the most abundant precursor ions from the survey scan (300–1,800 m/z) for HCD fragmentation. The automatic gain control (AGC) target was set to 3e6, and the maximum inject time was set to 10 ms. Dynamic exclusion duration was 40.0 s. Survey scans were acquired at a resolution of 70,000 at m/z 200 and resolution for HCD spectra was set to 17,500 at m/z 200, and the isolation width was 2 m/z. Normalized collision energy was 30 eV and the underfill ratio, which specifies the minimum percentage of the target value likely to be reached at maximum fill time, was defined as 0.1%. The instrument was run with the peptide recognition mode enabled.

### Protein identification and quantification

All MS raw data files were analyzed by Proteome Discoverer software 1.4 (ThermoFisher, U.S.A.) using the Mascot 2.2 search engine against a database of *mustela putorius furo* protein sequences (NCBInr, released March 23, 2017, containing 38, 992 sequences). For protein identification, a mass tolerance of 0.1 Da was allowed for fragmented ions, with permission of two missed cleavages in the trypsin digests: iTRAQ8-plex (Y), oxidation (M) as the potential variable modifications, and carbamidomethyl (C), iTRAQ8-plex (N-term), and iTRAQ8-plex (K) as fixed modifications. The strict maximum parsimony principle was performed, and only peptide spectra with high or medium confidence were considered for protein grouping. A decoy database search strategy was also used to estimate the false discovery rate (FDR) to ensure the reliability of the proteins identified.

For relative quantitation, proteins that involved at least one unique peptide were considered a highly confident identification and used for quantification. Additionally, to guarantee the accuracy of quantification, the proteins with coefficient of variation values <20% for three biological repeats were considered DEPs. The quantitative protein ratios were calculated and normalized by the median ratio in Mascot. For comparison, three identical mock samples, labeled with iTRAQ 113, 114, and 115, were used as references. Between samples, the proteins with fold-change ratios ≥1.20 or ≤0.83 and a *p* < 0.05 were considered DEPs according to the *t*-test.

### Bioinformatics analysis

To further explore the impact of the DEP on cell physiological processes and discover internal relations between DEPs, an enrichment analysis was performed. GO enrichment on three ontologies [biological process (BP), molecular function (MF), and cellular component (CC)] was applied based on the Fisher's exact test, considering the whole quantified protein annotations as the background dataset. Benjamini–Hochberg correction for multiple testing was further applied to adjust derived *p*-values. Only functional categories with *p*-values under a threshold of 0.05 were considered significant. KEGG pathway annotation was extracted from the online KEGG PATHWAY Database (http://www.kegg.jp/kegg/pathway.html).

The protein–protein interaction information involved in the immune response process of the studied proteins was subsequently retrieved from STRING software (http://string-db.org/). Then, the results were imported into Cytoscape5 software (http://www.cytoscape.org/, version 3.2.1) to visualize and further analyze functional protein-protein interaction networks.

### Real-time RT-PCR

Total RNA was isolated using TRIzol Reagent (Invitrogen, U.S.A.) from Mv.1.Lu cells infected with 2 MOI PS or mock-infected cells at 12 and 24 hpi. After treatment with gDNA Removal (TransGen Biotech, China), 4 μg of each total RNA was used for cDNA synthesis. Real-Time RT-PCR (qRT-PCR) assays were performed on an Applied Biosystems® QuantStudio® 3 System (Thermo Fisher Scientific, U.S.A.) employing the TransStart Top Green qPCR SuperMix kit (TransGen Biotech, China) according to the manufacturer's protocol. The primers for amplifying TRAF6, TRAF2, IRAK4, IRAK2, NFκB2, CCL2, TNF-α, IL-6, and GAPDH are presented in Table 1. Each experiment was performed in triplicate. The relative gene expression was calculated using the 2^−ΔΔCT^ model, which is representative of *n*-fold changes compared with mock-infected samples. The data was analyzed by two-way ANOVA followed by Duncan's test.

**Table 1:** Primers used for real-time RT-PCR.

**Name**	**Accession No**.	**Species**	**Primer sequence 5′-3′(Forward/Reverse)**	**Product size (bp)**
TRAF6	XM_004756001	Ferret	GAGAAACCCGTGGTCATT	194
			ATCGCAAGGCGTATTGTA	
TRAF2	XM_013058503	Ferret	GACGTGACCTCGTCCTCTTTC	192
			CCTGACTCCCAACCTGACCC	
IRAK4	XM_013063025	Ferret	TTCTTGCCCTGAGAACCA	191
			CTCCACTTTCCGATTTCC	
IRAK2	XM_004738517	Ferret	CTCACCGAGTACAGGAGC	162
			GAACTGCATCCAGTCCC	
NFκB2	XM_004749394	Ferret	TGAAGACCTTGCTGCTAAATG	112
			TCCAGGTTCTGTAAGGCTGTAT	
IL-6	EF368209	Ferret	CAACTATGAGGGTAATAAGAAC	194
			GCTCCGTAGGATGAGGTGAA	
CCL2	HAAF01015359	Mink	GAGGCTGACGAGCTAT	157
			AGTTTGGTTCTGGGTTT	
TNF-a	GU327784	Mink	GCCGACGTGCCAATGCCCTCCTG	223
			TCCCTTTGGCAAGGGCTCTTGAT	
GAPDH	NM_001310173	Ferret	GGTGCTGAGTATGTTGTGGAGT	197
			CAGTTGGTGGTACAGGAGGC	

### Western blot analysis

For testing the production of PS nucleoprotein for the different time points analyzed, cell lysates were harvested at 6, 12, 24, 36, 48, and 60 hpi from PS- and mock-infected samples. For confirmation of the iTRAQ-MS data by western blotting, cell lysates were harvested at 12 and 24 hpi from PS-, CDV_3_-, and mock- infected cultures. After measuring the protein concentrations, equivalent amounts of cellular proteins from the triplicates were separated by SDS-PAGE and electrophoretically transferred onto nitrocellulose PVDF membranes (Millipore, U.S.A.). The membranes were blocked with 2% BSA dissolved in TBS, containing 0.05% Tween-20, for 2 h at room temperature, followed by incubation with the corresponding primary antibodies (see below) at 4°C overnight and incubation with HRP-conjugated goat anti-rabbit or anti-mouse IgG secondary antibodies (Sangong Biotech, China) at room temperature for 2 h. The protein bands were detected using the ECL Detection Kit (Beyotime, China). The GAPDH protein was used as an internal control.

The following primary polyclonal antibodies were used: anti-CDV NP mouse monoclonal antibody (prepared in our laboratory), NF-κB p65 (RelA) rabbit polyclonal antibody (AN365, Beyotime, China), NFκB1 p105 rabbit polyclonal antibody (4717, CST, U.S.A), NFκBIB (IκB-β) rabbit polyclonal antibody (PA5-40909, ThermoFisher, U.S.A), MHC-I mouse monoclonal antibody (ab23755, Abcam, UK), RPS29 rabbit polyclonal antibody (PA5-41744, ThermoFisher, U.S.A), IκB-α rabbit polyclonal antibody (4812, CST, U.S.A), Phospho-NF-κB p65 rabbit polyclonal antibody (MA5-15181, ThermoFisher, U.S.A), and GAPDH rabbit polyclonal antibody (CW0101M, CWBIO, China).

### Immunofluorescence assay

Mv.1.Lu cells were cultivated on cover glasses in 24-well plates, followed by infection with PS or CDV_3_ at an MOI of 2 when the cells reached ~70% confluence. The mock-infected cells were treated with PBS as a negative control. Next, at 24 hpi, the cells were fixed with 4% paraformaldehyde and subsequently permeabilized with 0.1% Triton X-100. Further, the cells were incubated with an NF-κB P65 rabbit polyclonal antibody (Beyotime, China) and a mouse monoclonal antibody specific to CDV N protein and incubated with Cy3-labeled goat anti mouse IgG (Beyotime, China) and FITC-conjugated goat anti-rabbit IgG secondary antibody (ThermoFisher, U.S.A) prior to staining with DAPI. The fluorescent images were analyzed under confocal microscopy (Leica, Germany).

## Results

### Verification of PS replication in Mv.1.Lu cells

A previous study demonstrated the capacity of CDV growth in Mv.1.Lu cells (Lednicky et al., 2004), thus, we initially confirmed the ability of PS replication in Mv.1.Lu cells and established the growth kinetics of PS replication. An optimal time point under PS infection for proteomic analysis was then identified.

**Figure 1:**
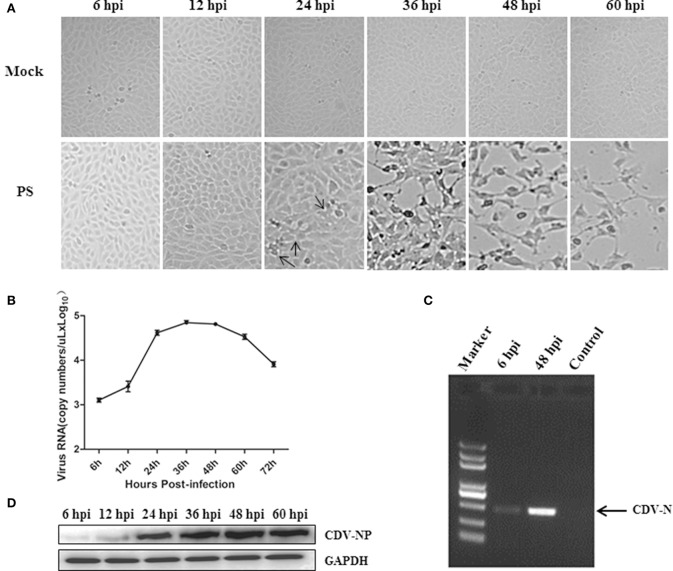
Confirmation of PS infection in Mv.1.Lu cells. **(A)** Photomicrographs of Mv.1.Lu cells infected with PS (MOI = 2) or mock-infected at different time-points. Images were taken at an original magnification of 20×. The CPEs of cells detachment at 24 hpi were pointed by the arrows. **(B)** One-step growth curve of CDV strain PS in Mv.1.Lu cells. **(C)** RT-PCR validation of PS infection in Mv.1.Lu cells by amplifying the CDV-N gene. **(D)** Western blot analysis of the nucleoprotein of PS in tested time points using anti-NP antibody.

As shown in Figure 1A, CPEs in the infection groups became visible at 24 hpi and progressed thereafter. Up to 36 hpi, an obvious CPE was observed and nearly 50 percent of the cells were detached at 48 hpi. The one-step growth curve revealed that the virus load reached a plateau of ~4.8 log_10_ copy numbers/μL between 24 and 60 hpi, followed by a gradual decline (Figure 1B). Collectively, 24 hpi was considered the optimal time-point for further proteomic analysis, at which a high viral load was maintained and most cells showed little CPE. Virus replication at 6 and 48 hpi was additionally ensured through RT-PCR. The abundance of the CDV-N gene increased as the infection progressed (Figure 1C). Further validation was performed by sequencing analysis of the PCR products (data not shown). Moreover, the production of nucleoprotein for the different time points analyzed was tested by anti-CDV NP antibody, the result showed quite similar tendency of the viral one-step growth curve (Figure 1D).

### Identification of differentially expressed proteins in PS-infected Mv.1.Lu cells

The host response to PS infection at 24 hpi was analyzed by examining differences in protein expression. Based on a combination of three biological replicates from mock-infected and PS-infected samples, the iTRAQ-coupled LC–MS/MS analysis identified and measured a total of 37,145 peptides and 6184 proteins. The proteins were designated DEPs based on the following criteria: a *p* < 0.05 and fold-change ratios ≥1.2 or ≤0.833. Among all the DEPs, 151 and 369 proteins were markedly up-regulated or down-regulated, respectively. Partial DEPs are shown in Table 2 and more detailed information for all DEPs is collated in Table S1.

**Table 2:** Partial differentially expressed proteins in Mv.1.Lu cells infected with PS.

**Accession No**.	**Gene**	**Description**	**Log2 ratios (infection/control)**
gi|511835322	C2orf78	Chromosome 2 open reading frame 78	4.41
gi|511926358	MHC-I	MHC class I	3.40
gi|511879956	ALDHLA3	Aldehyde dehydrogenase family 1, subfamily A3	3.09
gi|511896227	CBLC	Casitas B-lineage lymphoma c	2.53
gi|470656855	PPP4R4	Protein phosphatase 4, regulatory subunit 4	2.30
gi|511911718	VCAM1	Vascular cell adhesion molecule 1	2.23
gi|410968650	FMNL2	Formin-like 2	2.23
gi|511858686	IRAK4	Interleukin-1 receptor-associated kinase 4	2.08
gi|511851258	APOA1	Apolipoprotein A-I	2.03
gi|511858549	TSPAN8	Tetraspanin 8	2.00
gi|545550325	PKM	Pyruvate kinase, muscle	1.99
gi|511830126	IGFBP3	Insulin-like growth factor binding protein 3	1.98
gi|511841556	AHSG	alpha-2-HS-glycoprotein	1.96
gi|512014297	COL4A3	Collagen, type IV, alpha 3	1.95
gi|390460231	GPM6A	Glycoprotein m6a	1.95
gi|511845472	CCL2	Chemokine (C-C motif) ligand 2	1.95
gi|512003405	CDR2	Cerebellar degeneration-related 2	1.82
gi|297291910	RPS29	Ribosomal protein S29	1.80
gi|511836837	FAM71C	Family with sequence similarity 71, member C	1.73
gi|511894864	FN1	Fibronectin 1	1.73
gi|355716083	RelA	V-rel reticuloendotheliosis viral oncogene homolog A	1.71
gi|511888661	UGDH	UDP-glucose 6-dehydrogenase	1.70
gi|511829546	HUS1	Hus1 homolog	1.57
gi|511902668	DCLK1	Doublecortin-like kinase 1	1.56
gi|472388445	IRGM1	immunity-related GTPase family M 1-like	1.56
gi|511881226	ENO3	Enolase 3, beta muscle	1.56
gi|403261872	POU3F2	POU domain, class 3, transcription factor 2	1.55
gi|472347817	NINJ1	Ninjurin 1	1.52
gi|511875241	NSUN6	NOP2/Sun domain family, member 6	1.52
gi|511910703	KRT85	Keratin 85	1.52
gi|511859527	STX11	Syntaxin 11	1.49
gi|511902130	S100P	S100 calcium binding protein P	1.48
gi|511846797	ABCA1	ATP-binding cassette, sub-family A (ABC1), member 1	1.48
gi|6841210	ABRACL	costars family ABRACL	1.47
gi|511844818	B4GALT5	UDP-Gal: beta GlcNAc beta 1,4-galactosyltransferase, polypeptide 5	1.46
gi|511849816	TACO1	Translational activator of mitochondrially encoded cytochrome coxidase I	1.44
gi|511898473	RER1	RER1 retention in endoplasmic reticulum 1 homolog	1.43
gi|511903237	MAN1A2	annosidase, alpha, class 1A, member 2	1.43
gi|511908385	SH3BP5	SH3-domain binding protein 5 (BTK-associated)	1.42
gi|511943416	COMMD9	COMM domain containing 9	1.42
gi|511898597	TMEM68	Transmembrane protein 68	1.42
gi|511849632	APOH	Apolipoprotein H	1.42
gi|545185645	ARF1	ADP-ribosylation factor 1	1.41
gi|511935026	GBP6	Guanylate binding protein family, member 6	1.41
gi|512011829	EHD1	EH-domain containing 1	1.41
gi|533173825	PCP4	Purkinje cell protein 4	1.40
gi|511837380	GCAT	Glycine C-acetyltransferase	1.40
gi|512004618	NIT1	Nitrilase 1	1.39
gi|511870449	TRAF6	tumor necrosis factor receptor-associated factor 6	1.39
gi|511895854	SLC8A2	Solute carrier family 8, member 2	1.38
gi|512006423	REEP6	Receptor accessory protein 6	1.38
gi|511916720	UBE2L6	Ubiquitin ISG15-conjugating enzyme E2L 6	1.37
gi|511833334	USP48	Ubiquitin specific peptidase 48	1.37
gi|13775200	SF3B5	Splicing factor 3b, subunit 5	1.36
gi|511848426	TMCC3	Transmembrane and coiled coil domains 3	1.36
gi|511983423	ATP5D	ATP synthase, H+ transporting, mitochondrial F1 complex, delta subunit	1.35
gi|511921987	RBM15D	RNA binding motif protein 15B	1.35
gi|511868041	AP1G2	Adaptor protein complex AP-1, gamma 2 subunit	1.35
gi|511842841	FAM49A	Family with sequence similarity 49, member A	1.35
gi|511829942	SEMA3C	Sema domain, immunoglobulin domain	1.35
gi|511915046	CTSK	Cathepsin K	1.34
gi|511991226	SMS	Spermine synthase	1.34
gi|511869470	AEBP2	AE binding protein 2	1.34
gi|511832998	SFN	Stratifin	1.33
gi|30584771	TUBA4A	Tubulin, alpha 4a	1.33
gi|511882364	UNC13A	Unc-13 homolog A	1.33
gi|864509599	IL-6	Interleukin-6	1.33
gi|14210488	DCTN5	Dynactin 5 (p25)	1.32
gi|511831346	TNFAIP3	Tumor necrosis factor, alpha-induced protein 3	1.32
gi|511916377	CTSL2	Cathepsin L2	1.32
gi|119590561	HSPE1	Heat shock 10 kDa protein 1	1.32
gi|511883864	SUMF2	Sulfatase modifying factor 2	1.32
gi|545527366	NRBP1	Nuclear receptor binding protein 1	1.32
gi|511869866	ETV6	Ets variant gene 6 (TEL oncogene)	1.32
gi|511914585	FAM83G	Family with sequence similarity 83, member G	1.31
gi|355696495	IRAK2	Interleukin-1 receptor-associated kinase 2	1.31
gi|511904212	DUS3l	Dihydrouridine synthase 3-like	1.31
gi|511871618	PPP1R12B	Protein phosphatase 1, regulatory (inhibitor) subunit 12B	1.30
gi|511901047	C11orf68	UPF0696 C11orf68 homolog	1.30
gi|511906727	CNP	2′,3′-cyclic nucleotide 3' phosphodiesterase	1.30
gi|13385318	KDELR2	KDEL endoplasmic reticulum protein retention receptor 2	1.30
gi|511834309	BPGM	2,3-bisphosphoglycerate mutase	1.30
gi|511992880	GGH	Gamma-glutamyl hydrolase	1.30
gi|511825419	PDLIM7	PDZ and LIM domain 7	1.29
gi|511886519	WLS	Wntless homolog (Drosophila)	1.29
gi|511921959	RAD54L2	RAD54 like 2 (S. cerevisiae)	1.29
gi|332856788	PRMT1	Protein arginine N-methyltransferase 1	1.28
gi|511910087	LNP	Limb and neural patterns	1.28
gi|532072898	POLR3H	Polymerase (RNA) III (DNA directed) polypeptide H	1.28
gi|511914328	SAMD9l	Sterile alpha motif domain containing 9-like	1.27
gi|511833014	DHDDS	Dehydrodolichyl diphosphate synthase	1.27
gi|511974382	SERPINB2	Serine (or cysteine) peptidase inhibitor, clade B, member 2	1.27
gi|355707086	NFκB2	Nuclear factor of kappa light polypeptide protein enhancer in B-cells 2	1.27
gi|511923803	TNF-a	Tumor necrosis factor alpha	1.27
gi|511841350	PARL	Presenilin associated, rhomboid-like	1.27
gi|511862001	FOXO3	Forkhead box O3	1.27
gi|11345462	SPCS3	signal peptidase complex subunit 3	1.26
gi|511834349	CEP41	Centrosomal protein 41kDa	1.26
gi|511857535	HPS5	Hermansky-Pudlak syndrome 5	1.26
gi|511876736	PURG	Purine-rich element binding protein G	1.26
gi|511846480	GLIPR2	GLI pathogenesis-related 2	1.26
gi|511837127	C12orf23	UPF0444 transmembrane C12orf23 homolog	1.26
gi|355707083	NFκB1	Nuclear factor of kappa light polypeptide protein enhancer in B-cells 1	1.26
gi|511827086	FLNB	Filamin, beta	1.26
gi|511989679	LPCAT1	Lysophosphatidylcholine acyltransferase 1	1.26
gi|511876709	MAK16	MAK16 homolog (S. cerevisiae)	1.25
gi|511909041	CD2BP2	CD2 antigen (cytoplasmic tail) binding protein 2	1.25
gi|511827872	HMCES	RIKEN cDNA 8430410A17 gene	1.25
gi|511857770	MICAL2	Microtubule associated monoxygenase, calponin and LIM domain containing 2	1.25
gi|472384836	HSPG2	Heparan sulfate proteoglycan 2	1.25
gi|512007599	CLCN7	Chloride channel, voltage-sensitive 7	1.25
gi|511900977	YIF1A	Yip1 interacting factor homolog A	1.25
gi|472343859	CD47	CD47 antigen	1.25
gi|511983624	SCAMP4	Secretory carrier membrane protein 4	1.25
gi|511876596	WHSC1L1	Wolf-Hirschhorn syndrome candidate 1-like 1 (human)	1.25
gi|511850086	SH3PXD2A	SH3 and PX domains 2A	1.25
gi|511936255	TST	Thiosulfate sulfurtransferase (rhodanese)	1.25
gi|511911865	PHF11	PHD finger protein 11	1.25
gi|564300780	TRIM33	Tripartite motif-containing 33	1.22
gi|511902306	TAPBP	TAP binding protein (tapasin)	1.24
gi|511882769	MYO10	Myosin X	1.24
gi|511893223	KANK1	KN motif and ankyrin repeat domains 1	1.24
gi|511907397	MYL6B	Myosin, light polypeptide 6B	1.24
gi|511883719	TBL2	Transducin (beta)-like 2	1.24
gi|511884480	TOR3A	Torsin family 3, member A	1.23
gi|511873534	TRAF2	TNF receptor-associated factor 2	1.23
gi|555290040	AK6	Adenylate kinase isoenzyme 6	1.23
gi|511923928	ANO9	Anoctamin 9	1.23
gi|511902535	COL12A1	Collagen, type XII, alpha 1	1.23
gi|511887693	RAB38	RAB38, member of RAS oncogene family	1.22
gi|511906384	DHX8	DEAH (Asp-Glu-Ala-His) box polypeptide 8	1.22
gi|488526784	FCF1	FCF1 small subunit (SSU) processome component homolog	1.22
gi|511844820	PTGIS	Prostaglandin I2 (prostacyclin) synthase	1.22
gi|432094860	TUBA3A	Tubulin, alpha 3A	1.22
gi|27369539	RAP2C	RAP2C, member of RAS oncogene family	1.22
gi|511926986	GSTK1	Glutathione S-transferase kappa 1	1.22
gi|472384437	GOLPH3	Golgi phosphoprotein 3	1.21
gi|149017087	RPRD1A	Regulation of nuclear pre-mRNA domain containing 1A	1.21
gi|511908773	BCL2lL3	BCL2-like 13 (apoptosis facilitator)	1.21
gi|511841295	ATP11B	ATPase, class VI, type 11B	1.21
gi|511976077	AKAP2	Uncharacterized protein	1.21
gi|544446238	PRMT5	Protein arginine N-methyltransferase 5	1.21
gi|511976770	MNPP1	Multiple inositol polyphosphate histidine phosphatase 1	1.20
gi|511960500	P2RX4	Purinergic receptor P2X, ligand-gated ion channel 4	1.20
gi|511875437	TENM3	Teneurin transmembrane protein 3	1.20
gi|511901261	CDCA5	Cell division cycle associated 5	1.20
gi|512002654	TRABD	TraB domain containing	1.20
gi|511855781	TRMT6	tRNA methyltransferase 6 homolog (S. cerevisiae)	1.20
gi|511839811	E2F4	E2F transcription factor 4, p107/p130-binding	1.20
gi|511889285	EEF1A1	eukaryotic translation elongation factor 1 alpha 1	0.22
gi|511857546	L-LDH	L-lactate dehydrogenase	0.25
gi|511861258	RPH3A	Rabphilin 3A	0.27
gi|511866746	TDRD9	Tudor domain containing 9	0.33
gi|511911235	SMURF1	SMAD specific E3 ubiquitin protein ligase 1	0.37
gi|511974128	COL14A1	Collagen, type XIV, alpha 1	0.38
gi|511869618	MGP	Matrix Gla protein	0.39
gi|511836785	GATA6	GATA binding protein 6	0.4
gi|511837372	SH3BP1	SH3-domain binding protein 1	0.46
gi|511904960	CLDN25	Claudin 25	0.46
gi|512021328	WWC3	WWC family member 3	0.48
gi|511863285	ACSF3	acyl-CoA synthetase family member 3	0.49
gi|511976906	CCNT2	Cyclin T2	0.5
gi|511842520	CTDP1	CTD phosphatase, subunit 1	0.5
gi|511889359	SULT1C2	Sulfotransferase family, cytosolic, 1C, member 2	0.51
gi|511910161	C1orf123	Chromosome 1 open reading frame 123	0.51
gi|7657315	LSM3	LSM3-like protein, U6 small nuclear RNA associated	0.51
gi|511864485	DTD1	D-tyrosyl-tRNA deacylase 1 homolog (S. cerevisiae)	0.52
gi|431906893	KLF5	Kruppel-like factor 5	0.52
gi|511849400	NHE-RF	Na(+)/H(+) exchange regulatory cofactor NHE-RF	0.53
gi|257900516	REEP1	Receptor accessory protein 1	0.53
gi|472355383	RASA2	RAS p21 protein activator 2	0.54
gi|511898541	SKI	Ski sarcoma viral oncogene homolog (avian)	0.54
gi|511890018	CDK12	Cyclin-dependent kinase 12	0.55
gi|511910134	AGPS	Alkylglycerone phosphate synthase	0.56
gi|511883096	PPIC	Peptidylprolyl isomerase C	0.58
gi|511855302	CDAN1	Codanin 1	0.58
gi|511972404	SMTNL2	Smoothelin-like 2	0.59
gi|511845637	TADA2A	Transcriptional adaptor 2A	0.59
gi|511922352	EFEMP1	EGF containing fibulin-like extracellular matrix protein 1	0.59
gi|511893959	KIAA1671	RIKEN cDNA 2900026A02 gene	0.6
gi|511873494	CLIC3	Chloride intracellular channel 3	0.61
gi|511960748	ZFP592	Zinc finger protein 592	0.61
gi|472387045	PHPT1	Phosphohistidine phosphatase 1	0.62
gi|511876643	RAB11FIP1	RAB11 family interacting protein 1 (class I)	0.62
gi|472387045	PHPT1	Phosphohistidine phosphatase 1	0.62
gi|511903670	MKL1	Megakaryoblastic leukemia (translocation) 1	0.63
gi|511848742	LRRC45	Leucine rich repeat containing 45	0.65
gi|194211939	CACNB3	Calcium channel, voltage-dependent, beta 3 subunit	0.66
gi|511923560	INPP5J	Inositol polyphosphate 5-phosphatase J	0.66
gi|511915011	GABPB2	GA binding protein transcription factor, beta subunit 2	0.66
gi|511875266	DNAJC1	DnaJ (Hsp40) homolog, subfamily C, member 1	0.67
gi|511836048	LZTR1	Leucine-zipper-like transcriptional regulator, 1	0.67
gi|511849384	TMEM104	Transmembrane protein 104	0.67
gi|511838875	WDSUB1	WD repeat, sterile alpha motif and U-box domain containing 1	0.68
gi|555975747	NSA2	NSA2 ribosome biogenesis homolog (S. cerevisiae)	0.68
gi|511870441	PRR5L	Proline rich 5 like	0.68
gi|511829768	BCAP29	B cell receptor associated protein 29	0.69
gi|511913605	CCDC97	Coiled-coil domain containing 97	0.69
gi|301766733	FAM127A	FAM127-like	0.69
gi|511865423	NDFIP2	Nedd4 family interacting protein 2	0.69
gi|511881790	CCDC51	Coiled-coil domain containing 51	0.7
gi|511847011	ALAD	Aminolevulinate, delta-, dehydratase	0.7
gi|511833880	LZIC	Leucine zipper and CTNNBIP1 domain containing	0.7
gi|511903211	WARS2	Tryptophanyl tRNA synthetase 2, mitochondrial	0.7
gi|545881843	AGAP3	ArfGAP with GTPase domain, ankyrin repeat and PH domain 3	0.7
gi|511841170	PDLIM4	PDZ and LIM domain 4	0.71
gi|511913696	BLVRB	Biliverdin reductase B [flavin reductase (NADPH)]	0.71
gi|511906284	TMUB2	Transmembrane and ubiquitin-like domain containing 2	0.71
gi|511876053	ECHDC3	Enoyl CoA hydratase domain containing 3	0.71
gi|512011090	UPK3A	Uroplakin 3A	0.71
gi|355707095	NFκBIB	Nuclear factor of kappa light polypeptide protein enhancer in B-cells inhibitor, beta	0.72
gi|511896151	BLOC1S3	Biogenesis of lysosome-related organelles complex 1 subunit 3	0.72
gi|73950021	PTP4A2	Protein tyrosine phosphatase 4a2	0.72
gi|511883659	HSPB1	Heat shock protein 1	0.73
gi|511826747	CDH6	Cadherin 6, type 2, K-cadherin (fetal kidney)	0.74
gi|511854070	RHBDF1	Rhomboid 5 homolog 1 (Drosophila)	0.74
gi|512011195	LPP	LIM domain containing preferred translocation partner in lipoma	0.74
gi|511926830	DPP7	Dipeptidyl-peptidase 7	0.74
gi|511931993	LRP8	Low density lipoprotein receptor-related protein 8	0.75
gi|511862458	MTRF1L	Mitochondrial translational release factor 1-like	0.75
gi|511906536	COASY	bifunctional coenzyme A synthase isoform X3	0.75
gi|511890142	SP2	Sp2 transcription factor	0.75
gi|511910713	KRT7	Keratin 7	0.75
gi|511887578	TMEM126B	Transmembrane protein 126B	0.75
gi|281349685	ZFAND5	Zinc finger, AN1-type domain 5	0.75
gi|511853128	SRRM2	Serine/arginine repetitive matrix 2	0.75
gi|511986770	BDH2	3-hydroxybutyrate dehydrogenase, type 2	0.76
gi|545501819	TNRC6A	Trinucleotide repeat containing 6a	0.76
gi|511899033	TSC22D3	TSC22 domain family, member 3	0.76
gi|2286213	GNAQ	Guanine nucleotide binding protein, alpha q polypeptide	0.76
gi|511836121	MROPL40	Mitochondrial ribosomal protein L40	0.76
gi|511834186	NDUFB10	NADH dehydrogenase [ubiquinone] 1 beta subcomplex subunit 3	0.76
gi|511830220	PLA2G7	Phospholipase A2, group VII	0.76
gi|511896100	CD3EAP	CD3E antigen, epsilon polypeptide associated protein	0.76
gi|511907610	R3HDM2	R3H domain containing 2	0.76
gi|511843304	COL4A1	Collagen, type IV, alpha 1	0.77
gi|511878807	ELF1	E74-like factor 1	0.77
gi|511918709	UBQLN4	Ubiquilin 4	0.77
gi|511890619	SCFD1	sec1 family domain-containing 2	0.78
gi|511830488	CNPY3	Canopy 3 homolog (zebrafish)	0.78
gi|73965148	ARF2	ADP-ribosylation factor 2	0.78
gi|511909743	ZBTB10	Zinc finger and BTB domain containing 10	0.78
gi|511970268	CD2AP	CD2-associated protein	0.78
gi|511854253	SLC12A6	Solute carrier family 12, member 6	0.79
gi|511888766	LIMCH1	LIM and calponin homology domains 1	0.79
gi|511900684	SLC16A1	Solute carrier family 16, member 1	0.79
gi|511888312	GPAM	Glycerol-3-phosphate acyltransferase, mitochondrial	0.79
gi|511951833	PCM1	Pericentriolar material 1	0.79
gi|511848172	KANK2	KN motif and ankyrin repeat domains 2	0.79
gi|511925038	MRPS18B	Mitochondrial ribosomal protein S18B	0.79
gi|511913714	C19orf47	RIKEN cDNA 2310022A10 gene	0.79
gi|511943046	HS1BP3	HCLS1 binding protein 3	0.8
gi|511919532	NSL1	NSL1, MIND kinetochore complex component, homolog (S. cerevisiae)	0.8
gi|511906743	RABL3	RAB, member of RAS oncogene family-like 3	0.8
gi|511831382	REPS1	RalBP1 associated Eps domain containing protein	0.8
gi|511914923	RFX5	Regulatory factor X, 5 (influences HLA class II expression)	0.8
gi|511837364	GGA1	Golgi-associated, gamma adaptin ear containing, ARF binding protein 1	0.8
gi|511845503	RFFL	Ring finger and FYVE like domain containing protein	0.8
gi|511839695	CDH11	Cadherin 11, type 2, OB-cadherin	0.81
gi|511849128	EVPLl	Envoplakin	0.81
gi|511840215	WWP2	WW domain containing E3 ubiquitin protein ligase 2	0.81
gi|511866085	CKB	Creatine kinase, brain	0.81
gi|532066199	RPL10A	Ribosomal protein L10a	0.81
gi|511853705	CHTF18	CTF18, chromosome transmission fidelity factor 18	0.81
gi|511829476	FIGNL1	Fidgetin-like 1	0.81
gi|511975646	TFCP2	Transcription factor CP2	0.81
gi|511856781	CACUl	CDK2 associated, cullin domain 1	0.82
gi|511868239	JUB	Ajuba	0.82
gi|32880141	DNAJA1	DnaJ homolog subfamily A member 1	0.82
gi|511885805	STIM2	Stromal interaction molecule 2	0.82
gi|511916124	TCF12	Transcription factor 12	0.82
gi|345786001	NAA35	N(alpha)-acetyltransferase 35, NatC auxiliary subunit	0.82
gi|511897376	ARHGEF17	Rho guanine nucleotide exchange factor (GEF) 17	0.82
gi|511866734	AHNAK	AHNAK nucleoprotein isoform 1	0.83
gi|511856200	KANK4	KN motif and ankyrin repeat domains 4	0.83
gi|301762790	ZFP148	Zinc finger protein 148	0.83
gi|511849083	RHBPF2	Rhomboid 5 homolog 2 (Drosophila)	0.83

### Functional characterization of the DEPs

**Figure 2:**
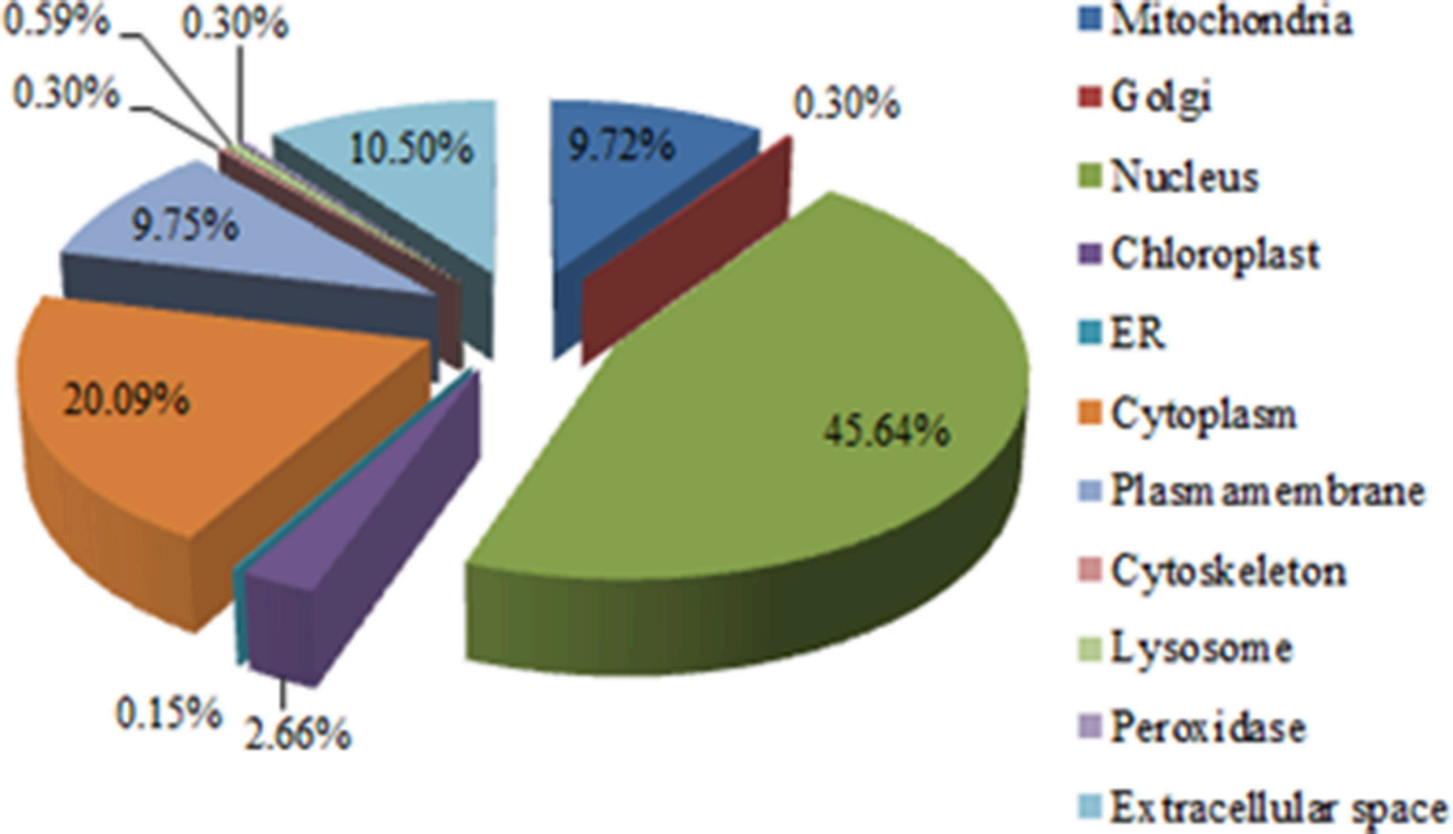
Subcellular localization of the DEPs in Mv.1.Lu cells infected with PS.

To characterize the biological functions of the 520 DEPs, canonical Gene Ontology (GO) enrichment were performed using DAVID (Dennis et al., 2003) and UniProt databases to obtain relevant annotations about the cellular components (CC), molecular functions (MF), and biological processes (BP). First, the putative subcellular localizations of the DEPs were analyzed. As depicted in Figure 2, a majority of the DEPs were mainly distributed in the nucleus (45.64%) and cytoplasm (20.09%), followed by extracellular space (10.50%), mitochondria (9.75%), and plasma membrane (9.72%), and a smaller portion were localized in the chloroplast (2.66%), lysosome (0.59%), Golgi (0.30%), cytoskeleton (0.30%), peroxidase (0.30%), and ER (0.15%) (more detailed information is collated in Table S2).

**Figure 3:**
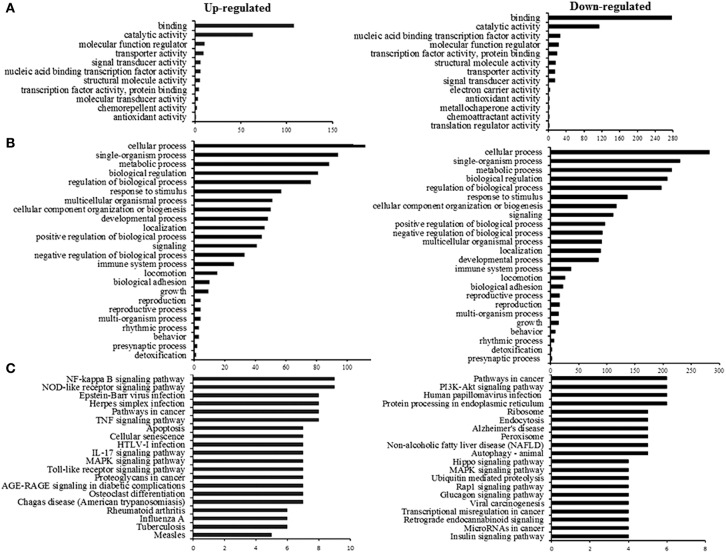
Functional characterization of the up-regulated and down-regulated proteins. **(A)** Molecular function (GO-MF) analysis. **(B)** Biological process (GO-BP) analysis. **(C)** KEGG Pathway analysis.

Interestingly, the GO analysis showed that most proteins were assigned to functions involved in similar molecular functions and biological processes. As shown in Figure 3A, most DEPs were closely related to binding and catalytic activity when infected by PS infection (more detailed information is provided in Table S3). The BP annotation showed that DEPs associated with various biological processes, including cellular process, metabolic process, biological regulation, immune system process and process of response to stimulus (Figure 3B) (more detailed information is provided in Table S4). Collectively, these categories consisted of the following proteins: CCL2, IRAK4, UBE2L6, NFκB1, NFκB2, TNF-a, IRAK2, IL-6, TRAF6, APOA1, TNFAIP3, TRAF2, RelA, and VCAM1 (up-regulated proteins) and CCR7, CXCR7, SMURF1, NFκBIB, MAPK7, RBM15, IGF2, TSC1, and CD59 (down-regulated proteins). To further investigate the pathways involving the identified DEPs, KEGG pathway analysis was performed. According to the results, DEPs were mainly involved in the NF-κB and NOD-Like receptor (NLR) signaling pathways. In addition, several proteins could be mapped to apoptosis and specific disease associations, consisting of infectious and respiratory diseases (Figure 3C) (more detailed information is shown in Table S5).

### Network analysis of the DEPs involved in immune response process

**Figure 4:**
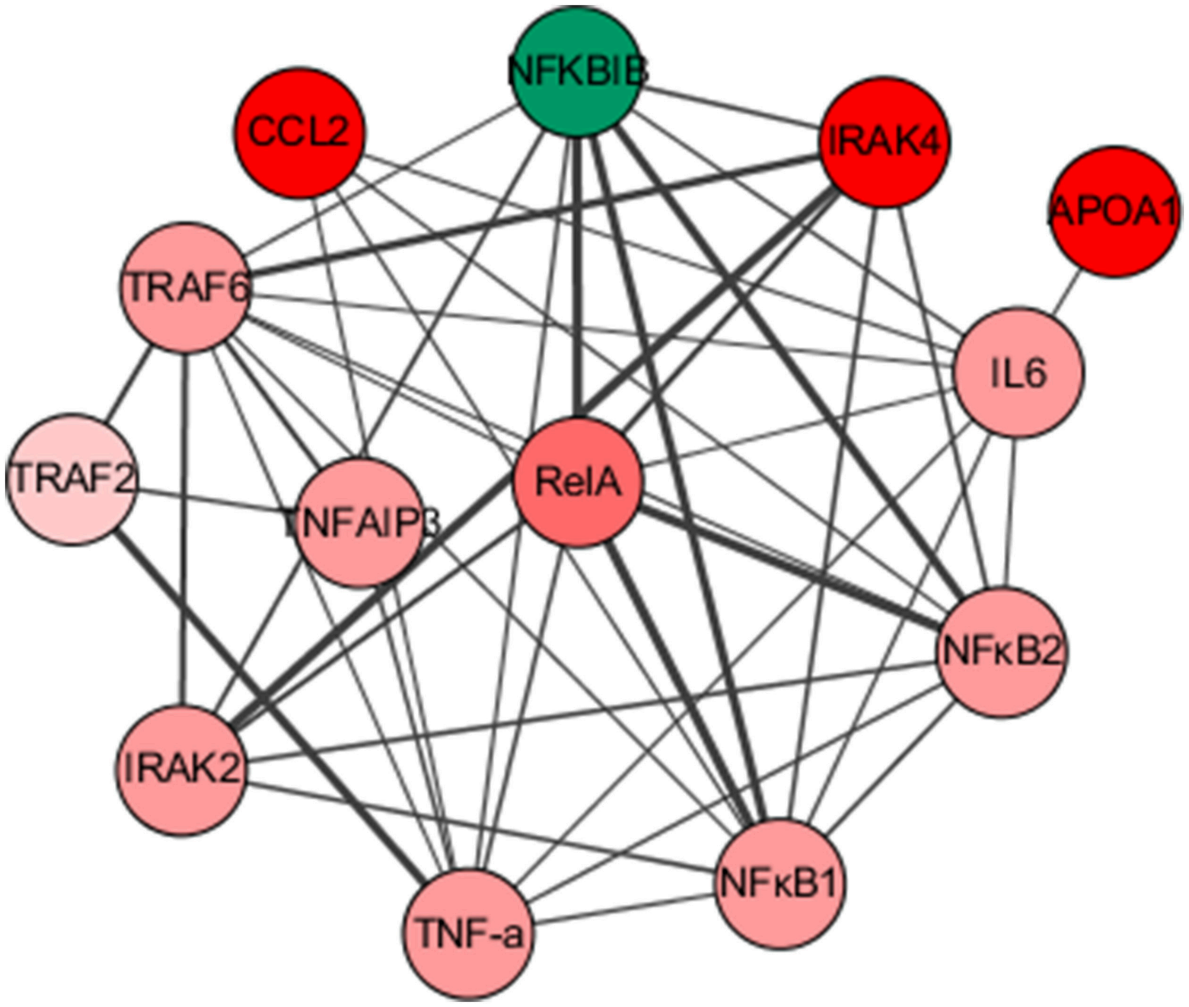
Network Analysis of the DEPs involved in immune response process. Significantly up-regulation and down-regulation proteins are represented in red and green, respectively. Varying magnitudes of the protein expression change are indicated in the color depth. The lines with different thicknesses showed the molecular relationships with different degree.

In the present study, we detected a total of 27 DEPs involved in the immune response process. To further investigate the interaction network associated with the immune response, these 27 proteins were imported into STRING software and further analyzed by Cytoscape5. As shown in Figure 4, 13 strongly interacting proteins were interestingly grouped into a functional set chiefly associated with the NF-κB signaling pathway. The interaction network provides clues for further illumination of the pathogenic mechanism and immunomodulation between CDV and the mink host.

### Confirmation of the iTRAQ-MS data by western blotting or real-time RT-PCR

**Figure 5:**
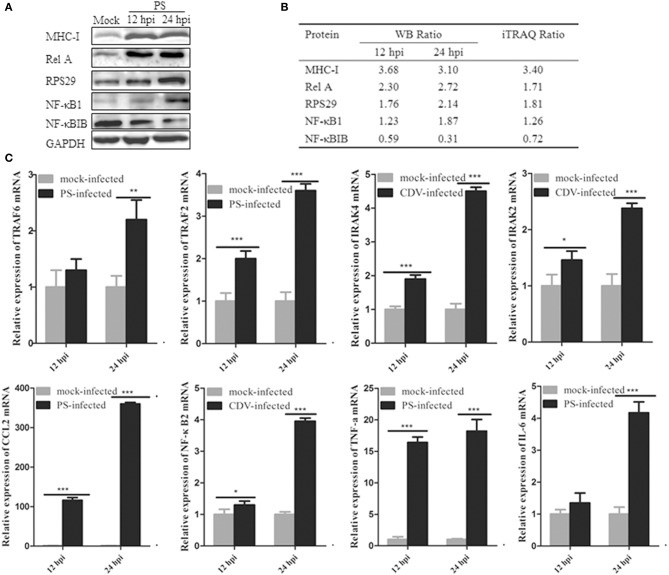
Confirmation of the iTRAQ-MS data by western blotting or real-time RT-PCR. **(A)** Western blot analysis of NF-κB1, RelA, MHC-I, RPS29, and NFκBIB in PS-infected and control samples at 12 and 24 hpi. GAPDH was served as internal reference. **(B)** The intensity ratio of the corresponding bands (infection/mock) was quantified using ImageJ software and normalized against GAPDH. **(C)** Eight selected differently expression proteins related to NF-κB pathway were testified using real-time RT-PCR method. Each gene was performed in three independent experiments. The relative gene expression was calculated using 2-^ΔΔ*CT*^ model, representative of *n*-fold changes in comparison with mock-infected samples. Error bars represent the standard error for triplicate samples. ^*^P < 0.05; ^**^P < 0.01; ^***^P < 0.001. The data was analyzed by two-way ANOVA followed by Duncan's test.

To confirm the iTRAQ-MS data, we selected significantly changed proteins, including NFκB1, RelA, MHC-I, RPS29, and NFκBIB, which reliably cross-reacted with polyclonal antibodies to the corresponding human proteins for western blotting analysis. As shown in Figure 5A, the five representative proteins showed up-regulated or down-regulated expression in PS-infected Mv.1.Lu cells at 12 and 24 hpi (the original blots are shown in Figure S1), in accordance with the results of the iTRAQ analysis (Figure 5B). However, due to the limitation of the availability of antibodies to *Neovison vison* proteins, the confirmation of DEPs by immunoblotting was restricted. Thus, eight other proteins involved in the immune response process were selected and tested using real-time RT-PCR. As illustrated in Figure 5C, compared to the mock group, mRNA expression of TRAF6, TRAF2, IRAK4, IRAK2, NFκB2, CCL2, TNF-a, and IL-6 in PS-infected cells was significantly up-regulated in a time-dependent manner, which further confirmed the iTRAQ-MS data.

### CDV infection induces the phosphorylation and nuclear translocation of NF-κB P65 and the degradation of IκB-α proteins

**Figure 6:**
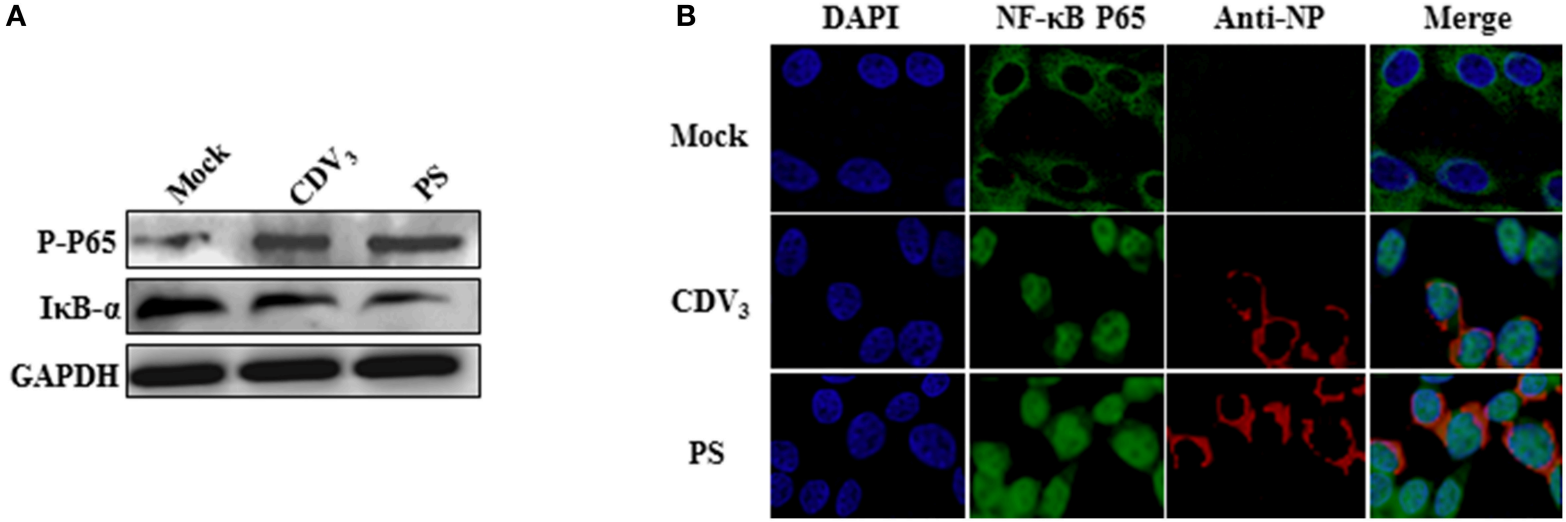
CDV infection induces the phosphorylation and nuclear translocation of NF-κB P65 and the degradation of IκB-α proteins **(A)** CDV infection strengthened NF-κB P65 phosphorylation and IκB-α degradation. Mv.1.Lu cells were infected with 2 MOI PS or CDV_3_. At 24 hpi, cells were gathered for detecting the expression levels of phosphorylated NF-κB P65 protein and IκB-α protein. **(B)** CDV infection facilitated NF-κB P65 nuclear translocation. Mv.1.Lu cells were infected with 2 MOI PS, CDV_3_ or mock-infected. At 24 hpi, the cells were fixed and incubated with rabbit polyclonal antibody specific to mink NF-κB P65 and mouse monoclonal antibody specific to CDV N protein, then incubated with FITC-labeled goat anti rabbit IgG and Cy3-labeled goat anti mouse IgG, respectively. Cell nuclei were stained by DAPI. The fluorescent images were analyzed under a confocal microscopy (Leica, Germany).

The activation of the NF-κB signaling pathway requires a series of cascade reactions, followed by the recruitment and phosphorylation of NF-κB protein and subsequent translocation from the cytoplasm to the nucleus, as well as the proteasome degradation of IκB proteins, which ultimately induces the production of inflammatory cytokines and type I IFN. Therefore, the degradation of IκB proteins (typically represented by IκB-α) and phosphorylation and nuclear accumulation of the NF-κB proteins (typically represented by NF-κB P65) are distinct features of NF-κB signaling pathway activation. The network analysis of the DEPs involved in the immune response has preliminarily indicated the induction of the NF-κB pathway by PS infection. To further validate this speculation, Mv.1.Lu cells were infected with PS at 2 MOI, after incubation for 12 or 24 h, total proteins were collected to measure the expression of IκB-a and phosphorylated NF-κB P65 proteins. As shown in Figure 6A, compared to that in mock-infected cells, phosphorylated NF-κB P65 (P-P65) and IκB-a proteins were obviously increased and decreased in PS-infected cells, respectively (the original blots are shown in Figure S2). To assess whether PS infection facilitates NF-κB P65 nuclear translocation, Mv.1.Lu cells were infected with PS at an MOI of 2 or mock infected for 24 h. As shown in Figure 6B, NF-κB P65 showed evident nuclear translocation in PS-infected cells but remained in the cytoplasm of mock-infected cells. Further, to determine whether other CDV strains could activate NF-κB P65, the expression of phosphorylated p65 and IκB-α was also detected in CDV_3_-infected cells, which was increased and decreased, respectively (Figure 6A). Additionally, the nuclear translocation of NF-κB P65 was also observed in CDV_3_-infected cells (Figure 6B).

## Discussion

CDV infection commonly causes a severe lethal disease in carnivores, including minks. However, the molecular mechanisms involved in viral pathogenesis and host immune responses have not been fully elucidated. To date, no research has focused on differential proteome analysis of host cells in response to CDV infection. Therefore, we utilized an iTRAQ approach to identify the DEPs to further explore the pathogenic mechanism and immunomodulation of CDV infection through an analysis of the effects on host cell proteins in the mink. The present study is the first to use Mv.1.Lu cells for iTRAQ analysis due to their ability to efficiently support CDV replication *in vitro*, and this cell line is homologous to the natural host system of minks.

As a starting point, we determined an optimal time to perform proteomic analysis by monitoring the CPEs and analyzing the one-step viral growth curve in PS-infected Mv.1.Lu cells. The results revealed that PS infection induced serials CPE changes from 12 to 60 hpi, with the virus load exhibiting a plateau between 24 and 60 hpi. Considering the high virus load was maintained at 24 hpi and most cells showed little CPE, we conducted the following proteomic analysis based on 24 hpi.

In total, we identified 151 up-regulated and 369 down-regulated proteins. Notably, an interesting observation in the present study was that CDV infection induces NF-κB activation in Mv.1.Lu cells. The NF-κB pathway regulates the expression of numerous immune system components to efficiently modulate the innate immune, inflammatory, and antiviral responses (Bose et al., 2003; Bours, 2005) and comprises a hub of cellular signal transduction pathways involved in host immune responses to viral challenge (Moynagh, 2005). So far, NF-κB has been reported as activated following various viral infections of porcine parvovirus (Cao et al., 2017), type 2 porcine circovirus (Wei et al., 2008), and herpes simplex type 1 (Patel et al., 1998). Additionally, NF-kB activation has previously been shown in MV infection (Helin et al., 2001) and was postulated as one of the mechanisms by which CDV might induce osteoclastogenesis (Mee and Sharpe, 1993). Moreover, NF-kB was subsequently demonstrated as induced by CDV (Onderstepoort strain) infection in human osteoclast precursors (Selby et al., 2006); however, these observations are all cases in humans or found in case of one single CDV strain. No reports of different CDV strains affecting NF-κB signaling in mink cells have been previously demonstrated. In the present study, nine NF-κB signaling regulators and downstream cytokines, including TNF-a, IRAK4, TRAF6, TRAF2, NFκB1, NFκB2, RelA, TNFaIP3, and VCAM1, were significantly up-regulated, and the NF-κB complex inhibitory protein IκB-β was obviously down-regulated. Further, KEGG pathway and network analyses of the DEPs involved in the immune response process also indicated the induction of the NF-κB signaling pathway. These results preliminarily indicated the activation of the NF-κB pathway by PS infection in Mv.1.Lu cells. More profound confirmation was observed by the detection of the phosphorylation and nuclear translocation of the NF-κB p65 subunit and the proteasome degradation of IκB-α protein in PS-infected Mv.1.Lu cells. Moreover, the activation of NF-κB p65 in CDV_3_-infected Mv.1.Lu cells also confirmed these findings. Together with the previous finding that NF-κB activation was found in human cells after CDV (Onderstepoort strain) challenge, these findings enriched the current knowledge of NF-κB activation by CDV infection, suggesting that NF-κB activation was not specific for a certain CDV strain or a certain species cells, but was suitable at least in part for several CDV strains and different species cells. Further validation is needed to compare the ability of various CDV strains to activate NF-κB signaling in other cell lines. In addition, some DEPs involved in the NF-κB pathway, containing IRAK4, RelA, TRAF6, NFκB1, and TNF-a together with IRAK2 and IL-6, were also identified as associated with measles and respiratory diseases, such as tuberculosis and pertussis, which are similar to the respiratory symptoms of CDV infection. The causative agent of measles is MV. In dogs and ferrets, CDV causes a disease that is highly similar to measles in humans (Hutchins et al., 2004; Perry and Halsey, 2004). Several theories have proposed that IL-6 is a critical inducer in the development of pagetic osteoclasts and bone lesions in Paget's disease induced by MV (Roodman et al., 1992; Ehrlich and Roodman, 2005). Mice expressing IL-6 and TNF-a in astrocytes suffer ataxia, inflammation and neurodegeneration after MV infection (Akassoglou et al., 1997; Raber et al., 1997). Therefore, the expression of these cytokines could contribute, in part, to mink pathological symptoms during CDV infection. Furthermore, in the present study, NLR signaling pathway was closely associated with PS infection. This innate immunity signaling pathway may play essential roles in the production of type I interferon and in promoting inflammasome assembly upon virus activation (Kobayashi et al., 2002; Sabbah et al., 2009). Recent studies have suggested that the inflammasome NLRP3, known as the NOD-like-receptor-family, pyrin domain-containing 3, recognizes several RNA viruses, such as influenza virus (Allen et al., 2009; Ichinohe et al., 2010), VSV (Rajan et al., 2011), and EMCV (Poeck et al., 2010). MV also activates the NLRP3 inflammasome, resulting in the caspase-1-mediated maturation of IL-1β (Zilliox et al., 2007; Komune et al., 2011). The NF-κB-induced activation of NLRP3 and pro-IL-1β gene expression is requisite for activating caspase-1 by the NLRP3 inflammasome to further regulate the secretion of the inflammatory cytokines IL-1β and IL-18 (Motta et al., 2015). However, whether there is signaling crosstalk between NF-κB activation and the NLR signaling pathway during CDV infection is an open question. Collectively, the findings suggested that activation of the innate immune NF-κB signaling pathway and the NLR signaling pathway was involved in mink immune responses against CDV infection, and the NF-κB signaling was associated with the pathological respiratory or other symptoms in mink after CDV infection. Further research may answer these questions.

CDV infection could cause gastrointestinal symptoms or severe diarrhea after secondary infection. The NHERF, Na^+^/H^+^ exchanger regulatory factor, commonly locates or becomes enclosed in the intestinal brush border, thereby binding to the renal proximal tubule brush border Na+/H+ exchanger NHE3 protein, which is mainly responsible for the absorption of electroneutral salt in the intestine and is the most essential sodium absorptive transporter (Donowitz et al., 2005). Therefore, NHERF plays a crucial part in establishing and maintaining the functional integrity of the intestinal barrier. Previous reports have demonstrated that NHERF down-regulation leads to reduced Na^+^ absorption though affecting NHE3 activity, ultimately increasing intestinal epithelial permeability and the risk of inflammatory bowel disease (IBD) (Sartor, 2006; Strober et al., 2007). Butler et al. discovered that the dysregulation of sodium transit contributed to piglet diarrhea and the pathogenicity of TGEV after infection (Butler et al., 1974). In the present study, NHERF is significantly down-regulated, consistent with a previous observation of the significant down-regulation of NHERF1 (a member of NHERF family) protein in TGEV-infected PK-15 cells using quantitative proteomic analysis (An et al., 2014). Accordingly, the observation suggested that the down-regulation of NHERF by PS infection induced disordered salt and water transit through NHE3 dysfunction and further leaded to in the malfunction of the sodium pump in the intestinal barrier, ultimately resulting in gastrointestinal symptoms or severe diarrhea in infected minks. The present study provides a new view of the pathogenesis of diarrhea in CDV-infected minks.

Ubiquitination, the covalent conjunction of ubiquitin to the target protein substrate, is the first of two successive steps associated with ubiquitin–proteasome pathway, which is responsible for a wide variety of cellular functions, including the activation of NF-κB signaling and type I IFN pathways (Ciechanover, 1994; Glickman and Ciechanover, 2002). Accumulated evidence has suggested that various viruses have evolved complicated mechanisms to exploit or manipulate the ubiquitin–proteasome pathway (Gao and Luo, 2006). For example, the activation of the ubiquitin–proteasome pathway is required for influenza virus replication (Widjaja et al., 2010) and is also required other viruses, such as rotavirus (Lopez et al., 2011), human cytomegalovirus (Tran et al., 2010), and porcine reproductive and respiratory syndrome virus (Zhou et al., 2014). The present study identified TRAF2, TRAF6, UBE2L6 (E2 ubiquitin ISG15-conjugating enzyme), USP48 (an ISG15 specific isopeptidase enzyme) and TRIM33 (E3 ubiquitin- ligase) as up-regulated proteins involved in protein ubiquitination. TRAF2 and TRAF6 are well-recognized as signal transducers in the NF-κB signaling pathway that function together with a dimeric ubiquitin-conjugating enzyme complex to catalyze the synthesis of K63-linked polyubiquitin chains and ultimately activate IκB kinase (IKK) and the downstream NF-κB pathway (Deng et al., 2000; Yang et al., 2016). As an IFN-induced ubiquitin-like protein, ISG15 plays a role in immunomodulation and imparting a direct antiviral activity against a wide spectrum of virus (Pincetic et al., 2010; Dai et al., 2011; Sooryanarain et al., 2017). Although the present study failed to detect the ISG15 protein, we identified the significantly up-regulated proteins UBE2L6 and USP48, which are strongly related to the ISGylation of ISG15. Similar to the mechanism of ubiquitination, ISGylation involves the sequential co-operation of E1, E2, E3 and an ISG15-specific isopeptidase enzyme (here identified as USP48) to facilitate ISG15 combination with target proteins for the execution of antiviral responses (Kroeker et al., 2013; Falvey et al., 2017). The tripartite-motif family (TRIM) of proteins plays essential roles in the innate immune responses to antimicrobial infections. TRIM33, a member of the TRIM family and previously known as transcriptional intermediary factor 1 gamma (TIF1-γ), functions in monocyte/macrophage mediated inflammation (Gallouet et al., 2017) and inflammasome activation (Weng et al., 2014). Our results provided the first evidence of multiple differentially up-regulated immune-related proteins associated with protein ubiquitination in response to PS infection in Mv.1.Lu cells, indicating that ubiquitination appeared to be a pivotal regulatory mechanism in the immune responses to CDV infection in mink.

Apoptosis plays a role in regulating the pathogenesis of various infectious diseases, which oppositely affect viral pathogenesis by either restraining viral transmission or accelerating viral propagation by the release of the virus particles (Pastorino et al., 2009). In the present study, seven up-regulated proteins, including TNF-a, RelA, NFκB1, TRAF2, a-tubulin, CTSK (Cathepsin K), and CTSV (Cathepsin V), were identified as apoptosis-related, suggesting the induction of apoptosis in PS infection in Mv.1.Lu cells. CTSK and CTSV are associated with a mitochondria-dependent intrinsic pathway to trigger the apoptosis of host cells, while TNF-a participates in an extrinsic receptor-mediated pathway (Benedict et al., 2002). This finding was consistent with previous reports showing that CDV induces apoptosis in the cerebellum and lymphoid tissues of the natural infection of dogs and in Vero cells *in vitro* (Moro et al., 2003; Del Puerto et al., 2010, 2011). The mechanisms of apoptosis in the pathogenesis of CDV have not yet been clearly illuminated, and the extensive study of these proteins should enhance the current understanding of the mechanisms underlying apoptosis regulation during CDV infection.

In summary, the present study provides the first overview of the protein alterations in CDV-infected Mv.1.Lu cells using iTRAQ analysis. The identification of differently expressed proteins reflects a comprehensive interaction network of Mv.1.Lu cells and CDV during infection. Although some significantly regulated proteins were suggested to be related to the pathological symptoms and the immune responses to CDV infection, further functional elucidations are needed to clarify the pathogenic mechanisms and the immune responses to additionally identify new therapeutic targets for preventing CDV infection.

## Author contributions

MT and SC designed the study; MT, LY, NS, and YC performed the experiments; ZC and JW analyzed the data; SL, PL, and YS prepared the figures and tables; MT wrote the manuscript.

### Conflict of interest statement

The authors declare that the research was conducted in the absence of any commercial or financial relationships that could be construed as a potential conflict of interest.
