# Supplementary figures and images for: Quantitative Analysis of Cellular Proteome Alterations in CDV-Infected Mink Lung Epithelial Cells
Source: Front Microbiol. 2017 Dec 22;8:2564. doi: 10.3389/fmicb.2017.02564 (PMC5743685; doi:10.3389/fmicb.2017.02564)

## Slide 1
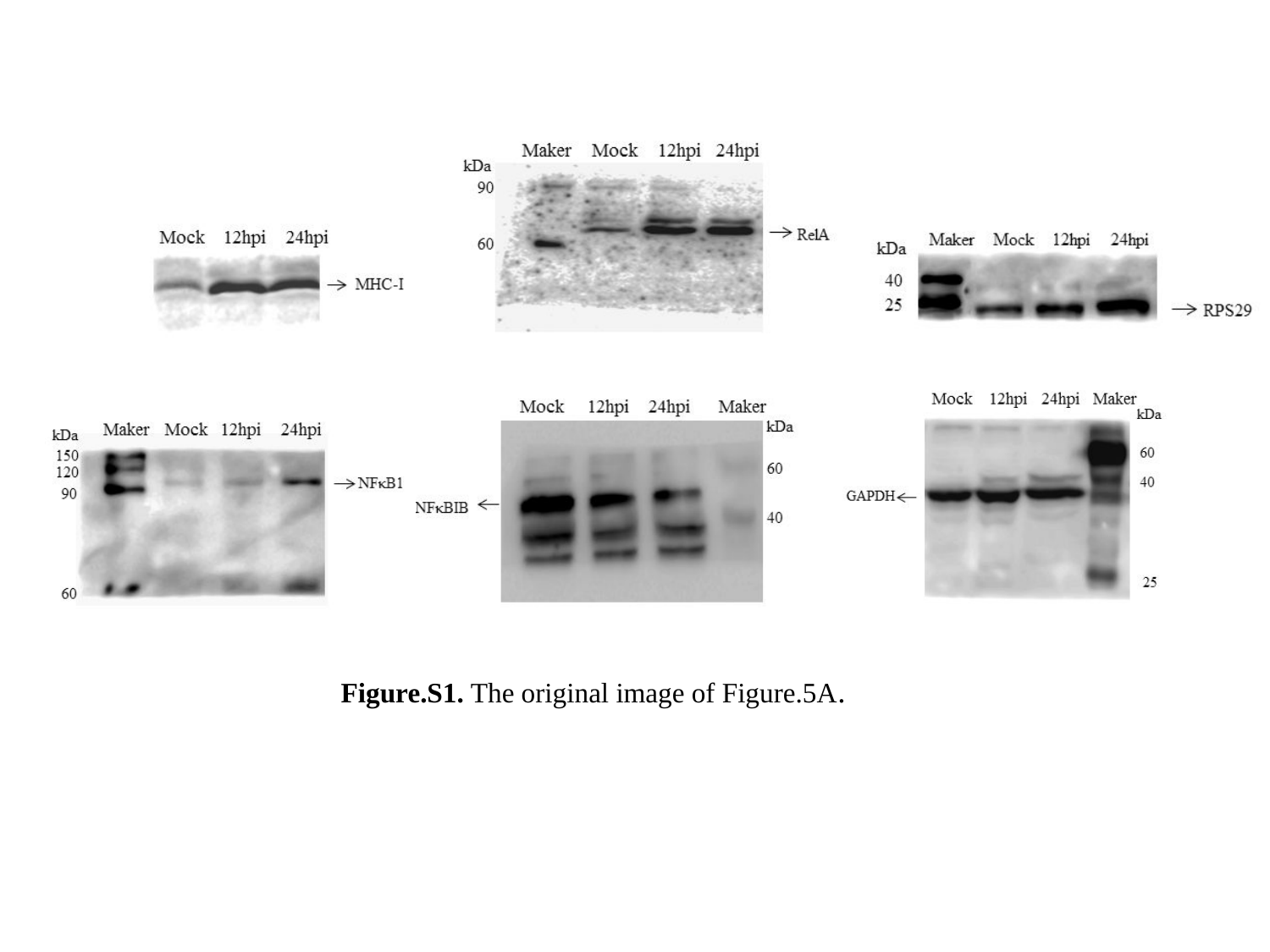

Figure.S1. The original image of Figure.5A.

Supplement: Supplementary file 6 [file Presentation1.PPTX]

## Slide 1
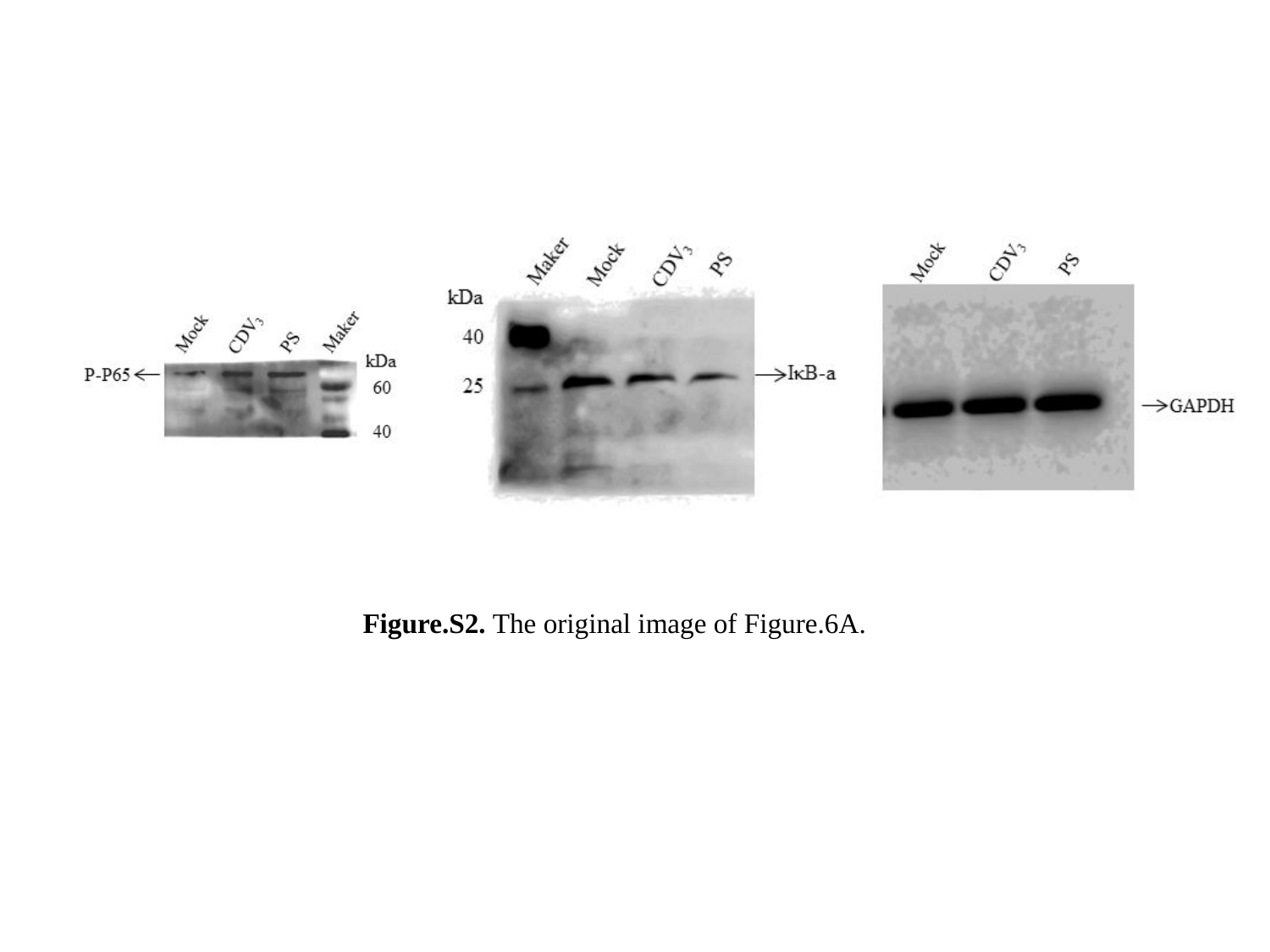

Figure.S2. The original image of Figure.6A.

Supplement: Supplementary file 7 [file Presentation2.PPTX]
